# First-episode psychosis in treatment-resistant schizophrenia: a cross-sectional study of a long-term follow-up cohort

**DOI:** 10.1186/s12888-018-1853-1

**Published:** 2018-09-03

**Authors:** Nobuhisa Kanahara, Hiroshi Yamanaka, Tomotaka Suzuki, Masayuki Takase, Masaomi Iyo

**Affiliations:** 1 Division of Medical Treatment and Rehabilitation, Center for Forensic Mental Health, Chiba University, 1-8-1 Inohana, Chuou-ku, Chiba-shi, Chiba, 260-8670 Japan; 2 Department of Psychiatry, Chiba University Graduate School of Medicine, 1-8-1 Inohana, Chuou-ku, Chiba, 260-8670 Japan; 3 Department of Psychiatry, Chiba Psychiatric Medical Center, 5 Toyosuna, Mihama-ku, Chiba 261-0024 Japan; 4 Department of Psychiatry, Koutoku-kai Sato Hospital, 948-1 Kunugizuka, Nanyo City, Yamagata 999-2221 Japan; 5 Kokoro Clinic Monzen-nakacho, Ikkou Building 1F, 1-3-5 Tomioka, Koutou-ku, Tokyo, 135-0047 Japan; 6 Division of Psychiatry Research, The Zucker Hillside Hospital, 75-59 263rd St, Glen Oaks, NY 11004 USA

**Keywords:** Antipsychotic, First-episode psychosis, Negative symptoms, Positive symptoms

## Abstract

**Background:**

Approximately one-third of schizophrenia patients eventually develop treatment-resistant schizophrenia (TRS). Although the time course of TRS development varies from patient to patient, the details of these variations have not been clarified. The present study compared the duration of time required to achieve control of the first-episode psychosis (FEP) between patients who went on to develop TRS and those who did not, in order to determine whether a bifurcation point exists for the transition to TRS.

**Methods:**

The present study included 271 schizophrenia patients. Based on the clinical assessment, each patient was assigned to a TRS (*n* = 79) or Non-TRS group (*n* = 182). Clinical factors relating to FEP treatment such as the duration of initial hospital admission and the degree of improvement were retrospectively identified.

**Results:**

There was no significant difference in the duration of initial hospital admission (defined as the time from treatment introduction to successful discharge) between the two groups (mean of 87.9 days for TRS vs. 53.3 days for Non-TRS). The degree of improvement during initial hospital admission of the TRS group was significantly lower than that of the Non-TRS group (Global Assessment of Functioning (GAF) of 50 points for TRS vs. 61 points for Non-TRS). Approximately half of the TRS patients showed an acute onset pattern and longer hospital admission (mean 169 days) for their FEP. The other half of TRS patients needed no hospital admission, indicating an insidious onset pattern with no clear psychotic episode and treatment introduction without hospital admission.

**Conclusions:**

Future TRS patients can have difficulty in improvement during their FEP. There appear to be two distinct patterns for the development of TRS. One pattern is characterized by refractory positive symptoms and a longer period to control the first psychosis; the other shows latent or insidious onset and poor response to the initial treatment.

## Background

Schizophrenia is a severe mental disorder encompassing a variety of psychiatric symptoms including positive symptoms such as delusion and hallucination, negative symptoms such as avolition and social withdrawal, and further cognitive impairments and mood dysregulation. Pharmacological medication has been a mainstay in treating patients with the disease, but only one-third of patients successfully recover to their premorbid functioning level without any significant psychotic symptoms; the majority of patients have some remaining symptoms, and their symptoms are often exacerbated during the long-term disease course [1]. In clinical practice, such recurrent episodes often exhibit insufficient or no response to antipsychotics.

The reasons why some patients who respond well to treatment for their first-episode psychosis (FEP) subsequently develop treatment-resistant schizophrenia (TRS) are not well understood. Several clinical trials have reported that approximately 15% of patients with schizophrenia show no response to the first antipsychotic in their FEP, whereas 75% of FEP patients show a significant response [2–4]. On the other hand, it has been estimated that about 30–40% of all patients eventually fulfill the criteria of TRS [5, 6]. These data suggest that among patients eventually classified as having TRS, a few demonstrate TRS at an early treatment phase while the rest transit into TRS at various later time-points during their disease course.

Recent studies have suggested that there may be two patterns of development to TRS: an immediate transition to TRS from the time of treatment introduction (i.e., early TRS) and a later transition to TRS after a significant improvement (i.e., later TRS) [7, 8]. In addition, younger onset, higher initial negative symptoms and longer duration of untreated psychosis (DUP) have been proposed as factors predictive of transition to TRS [8]. Despite these findings, however, the detailed process of development to TRS remains to be elucidated. For example, it is unclear whether patients who meet the criteria of early TRS will continue to meet the TRS criteria thereafter. In order to more clearly clarify the process to TRS and to exclude, to the greatest degree possible, any ambiguity in the diagnosis of TRS, we considered that it would be reasonable to focus exclusively on patients undergoing long-term treatment who were currently diagnosed with TRS. Therefore, we decided to retrospectively compare duration and clinical factors of FEP between current TRS patients and non-TRS patients. We hypothesized that subjects with current TRS would have previously exhibited poorer improvement during treatment of their FEPs compared to subjects without TRS. To examine this, we compared the duration of initial hospital admission, which we defined as the period from treatment introduction to successful discharge (with successful discharge defined as a subsequent 3 months of successful treatment in an outpatient clinic), among subjects with and without current TRS.

## Methods

### Subjects

**Fig. 1:**
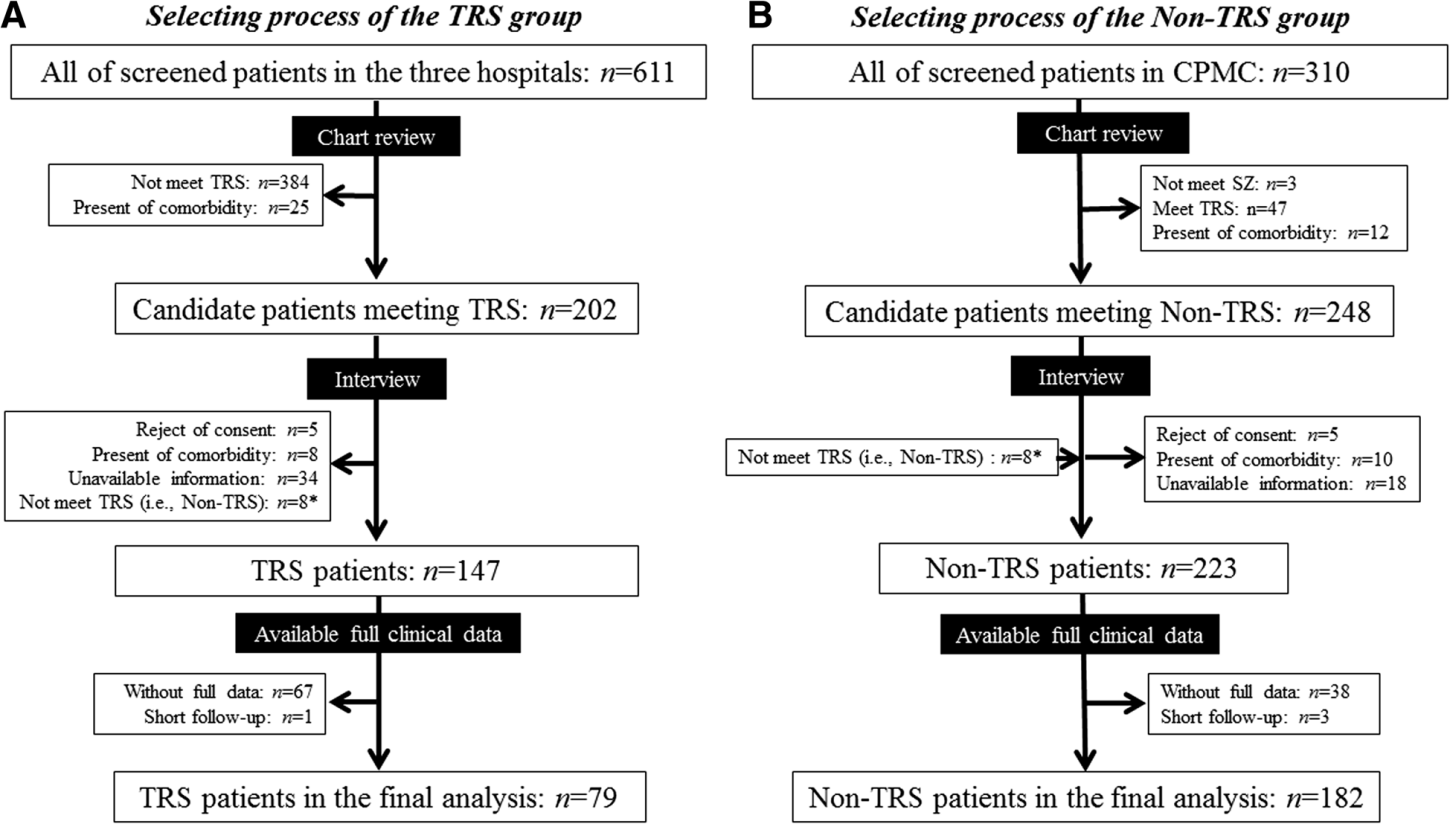
Overview of participant flow. **a** Selection process for the TRS group. The study cohort consisted of patients treated at three facilities. **b** Selection process for the Non-TRS group. The Non-TRS group consisted mainly of patients from the Chiba Psychiatric Medical Center (CPMC). *These 8 subjects were not judged as having TRS, and were therefore included in the Non-TRS group. The process used for the selection of the study subjects is described in detail in Yamanaka et al. [10]

We reviewed the medical charts of 611 patients with a diagnosis of schizophrenia or schizoaffective disorder (DSM-IV-TR) who were treated in any of three psychiatric facilities in Japan from April 2012 to September 2014 (Fig. 1).

From among them, we selected 202 patients who were candidates for a diagnosis of TRS. For purposes of the present study, we performed an in-person interview for each of the patients. Diagnosis of TRS was judged based on the combined criteria of the Clozaril Patients Monitoring Service (CPMS) and the Broadest Eligibility Criteria [5]. That is, if a patient’s psychotic symptoms did not respond sufficiently to two different classes of antipsychotics with a 600 mg or greater chlorpromazine-equivalent (CP-eq) dose for at least 4 weeks and concurrently his/her Global Assessment of Functioning (GAF) score did not exceed 61 points on average within 1 year prior to the study screening, then he or she was diagnosed with TRS as a “poor responder”. If a patient’s extrapyramidal symptoms could not be controlled well by anti-parkinson agents, he or she was diagnosed with TRS for showing “intolerance to antipsychotics”. Patients who met the “poor responder” criterion at any past time point, but who did not meet this criterion at the study interview, were classified into the Non-TRS group. As result, 147 patients were classified into the TRS group. Of them, subjects who were treated for short-term duration or whose full medical record was unavailable were excluded. Thus the final TRS group used for the analysis consisted of 79 patients.

Clozapine was introduced in mid-2009 in Japan, and was available in only one study facility (Chiba University Hospital) during the study period. Several patients gave their informed consent to undergo clozapine treatment and participated in the present study interview prior to clozapine introduction. Because they had not yet been treated with clozapine, these patients were not excluded from the TRS group.

For the Non-TRS group, a total of 248 subjects were selected from 310 subjects treated in a single facility (Chiba Psychiatric Medical Center: CPMC), and then through the interview process, 223 of these subjects were classified as non-TRS patients (Fig. 1). Forty-one patients for whom full clinical data were not available were excluded, resulting in a final Non-TRS group of 182 patients. Patients whose primary diagnosis was any axis I or II disorder were excluded from the study; 37 subjects were excluded by this criterion, all of them due to mental retardation or pervasive developmental disorder. Subjects with a history of illegal drugs were not excluded from the present study, but there were few patients with illegal drug usage as a comorbidity.

The diagnosis of TRS was performed as accurately as possible based on the interview of each of 20 potential TRS patients by at least two of the four researchers (N.K., H.Y., T.S., and M.T.); each of the researchers underwent several training sessions and were required to reach a consensus in their diagnosis [9]. The reason for the relatively high dropout of subjects, particularly in the TRS group, was that a significant portion of the TRS subjects treated in two of the participating facilities (Chiba University Hospital and CPMC) were transferred to their local psychiatric hospital for a long hospital stay, since these facilities do not provide long-term care.

The ethical review boards of the three participating hospitals approved the present study, and written informed consent was required from each patient or his/her guardian upon being given a detailed explanation of this study.

### Assessments

#### Factors related to the treatment of first-episode psychosis

**Fig. 2:**
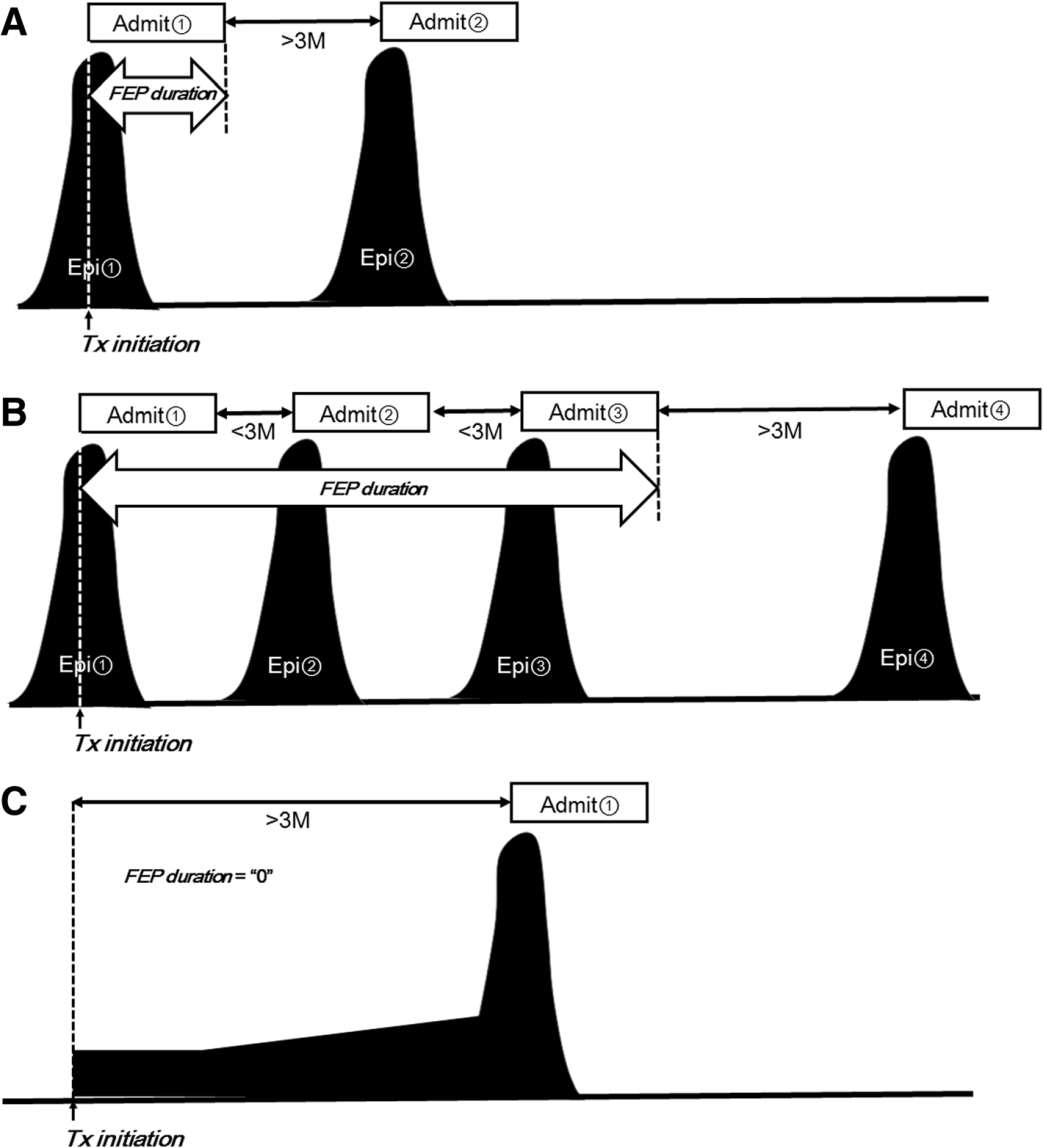
The method of determining the duration of first-episode of psychosis (FEP) in the present study. **a** A sample case of a patient who first visited the hospital and was admitted on the same day. If this patient was discharged and did not require readmission within 3 months, the FEP duration would be evaluated as the period from the date of the initial hospital admission to the date of discharge from the indexed admission. **b** A sample case of a patient starting medication in the inpatient setting as in the case of A), but requiring hospital readmission within 3 months following the date of the indexed admission. The FEP duration is evaluated from the date of the initial hospital admission to the date of discharge (the third discharge) (that was *not* followed by readmission within 3 months). **c** A sample case of a patient who did not require hospital admission for FEP treatment within 3 months following the medication onset. This FEP duration is evaluated as “0”

The primary measure of the present study was the duration of initial hospital admission, which was defined as the duration from the first hospital admission to the achievement of clinical stability based on the medical records. Specifically, as shown in Fig. 2, if a given patient started medication for FEP at hospital admission, the duration of initial hospital admission was calculated from the date of hospital admission to the date of successful discharge from the hospital (with successful discharge defined as an improvement of symptoms and no requirement of hospital readmission for at least 3 months) (A). If a patient started medication at an outpatient clinic and was later admitted to the hospital within 3 months from the treatment introduction, the measure was calculated from the date of the introduction at the outpatient clinic to the date of successful discharge from the hospital (improvement in symptoms; not requiring hospital readmission for at least 3 months) (B). If a patient started medication at the outpatient clinic and further continued the medication there for more than 3 months, the measure was considered to be zero (C).

Regarding measurements of the responsiveness to FEP treatment, antipsychotic dosage and GAF were determined in the following manner. In patients starting their treatment at the hospital admission, these measures were defined at the discharge from the hospital for subjects who were discharged within 6 months from admission, and at the 6th month for those whose admission lasted more than 6 months. In patients starting at the outpatient clinic, these measures were defined at the date when antipsychotic(s) had remained the same for at least 4 weeks within 6 months from the treatment introduction, or were defined at 6 months for a subject whose antipsychotic(s) were never established within the first 6 months.

#### Factors prior to treatment introduction

The following factors were identified from the medical records. (1) age at onset, (2) DUP, and (3) premorbid social adjustment level; these were defined in the generally accepted manner as described in our previous studies [9, 10]. (4) The mode of onset (MoO) was defined as “acute onset” when the period from the appearance of prominent deterioration in daily functioning and/or any psychotic symptom to a behavioral problem requiring the first hospital admission was 3 months or less, and “insidious onset” when this interval was longer than 3 months. This “3-month rule” was longer than that of other similar studies [11, 12].

All of the information prior to treatment introduction, including the data on MoO, were obtained from the medical records generated for each patient after his/her first visit. Most of these records were written in a fair amount of detail, based on interviews with the patient, his/her family and sometimes police officers or the staffs of administrative agencies. A preliminary search of these documents prior to the present study revealed only a few cases in which psychosis occurred within 1 month, which is the formal definition of acute onset. On the other hand, in many cases a patient’s family members observed close-hand that the patient experienced a relatively long process of development of psychosis. For these reasons, we applied the “3-month rule” to catch all cases with insidious onset in the present study.

### Statistical analysis

We used SPSS ver. 19.0 (IBM, NY, USA) for the statistical analysis in the present study. For comparisons between the groups, Student’s *t*-test was applied for continuous values, and chi-square test or Fisher’s exact test was applied to categorical values. The threshold level of significance was set at α = 0.05.

## Results

### The TRS group vs. non-TRS group

There were no differences in age or gender between the TRS and Non-TRS groups (Table 1).

**Table 1:** Demographic and clinical characteristics of the TRS Group and Non-TRS Group

Variables	TRS Group *n* = 79	Non-TRS Group *n* = 182	Statistic Values
*General information*			
Age (years)	41.8 [11.1]	43.9 [11.5]	*t* = 1.375, *P* = 0.170
Sex: Male/Female (n)	47/32	98/84	Chi = 0.712, *P* = 0.400
Follow-up duration (days)	6139.7 [3692.6]	4477.8 [2269.7]	*t* = −3.708, ***P*** **< 0.001**
Duration before meeting TRS (days)	3195.2 [3148.8]	–	
*Clinical index prior to treatment introduction*			
Age at onset (years)	24.0 [8.30]	29.4 [9.63]	*t* = 4.306, ***P*** **< 0.001**
Premorbid social adjustment	4.71 [3.15]	3.61 [2.88]	*t* = −2.723, ***P*** **= 0.007**
DUP (months)	9.61 [16.5]	21.9 [47.8]	*t* = 1.976,
DUP median	3.00	3.75	***P*** **= 0.049**
Mode of onset: Acute/Insidious (n)	38/41	74/108	Chi = 1.245, *P* = 0.264
*Clinical index related to FEP*			
Duration of initial hospital admission (days)	87.9 [205.6]	53.3 [49.4]	*t* = −1.476, *P* = 0.144
GAF after FEP	50.1 [16.6]	61.0 [13.0]	*t* = 5.176, ***P*** **< 0.001**
GAF > 70 (n)	11	52	Chi = 6.454, ***P*** **= 0.011**
CP-dose for FEP treatment (mg)	562.4 [410.5]	703.4 [437.6]	*t* = 2.405, ***P*** **= 0.017**

*Abbreviations*: *CP* chlorpromazine, *DUP* duration of untreated psychosis, *FEP* first-episode psychosis, *TRS* treatment-resistant schizophrenia

#### Duration of initial hospital admission

**Fig. 3:**
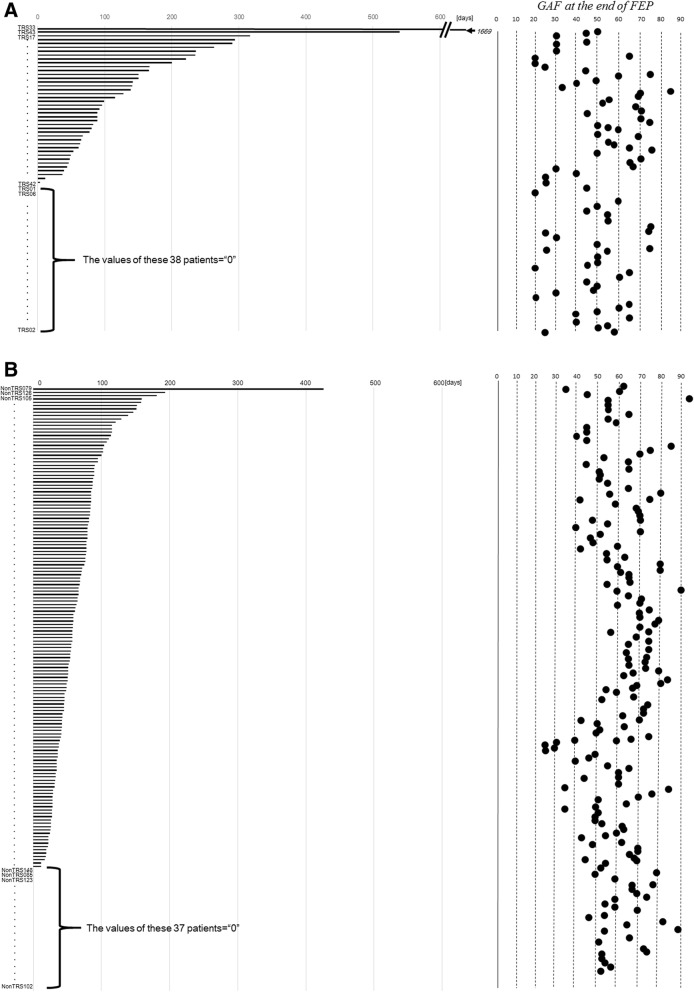
FEP durations and GAF at the end of FEP in the TRS and Non-TRS groups. **a** The TRS group. **b** The Non-TRS group. Patients were sorted by FEP duration, as indicated by the horizontal axis

The duration of initial hospital admission-the time from treatment introduction until successful discharge (no rehospitalization within 3 months)- was longer in the TRS group than in the Non-TRS group, but the difference did not reach statistical significance. However, significant differences in the factors related to treatment during the initial hospital admission were observed between the TRS and Non-TRS group. Namely, patients in the TRS group had lower GAF scores and lower drug doses than the patients in the Non-TRS group. GAF scores of 70 points or greater, indicating a good functioning level, were observed in 11 (13.9%) patients in the TRS group and 52 (28.6%) patients in the Non-TRS group (Table 1 and Fig. 3).

#### Other measures

Regarding baseline factors prior to treatment introduction, although the MoO did not differ between the two groups, age at onset and premorbid social functioning of the TRS groups were significantly younger and lower, respectively, compared to the Non-TRS group. The DUP of the TRS group was marginally shorter than that of the Non-TRS group. The number of hospital admissions over the follow-up period and the admission duration following the initial hospital admission in the TRS group were higher and longer, respectively, compared to those in the Non-TRS group.

### TRS subgroup comparison: The TRS patients with admission vs. without admission for FEP

When patients were sorted according to the length of their initial hospital admission, there were a significant number of patients in the TRS group with an FEP duration of “0” (38/78 = 48.1%: Fig. 3a). A value of 0 in this measure indicates treatment in an outpatient clinical setting without hospital admission within the first 3 months. Based on this finding, we decided to divide the TRS group into two subgroups, those with admission for FEP (duration of initial admission > 0) and those without admission (duration of initial admission = 0) within 3 months following treatment introduction, and the data for these subgroups were analyzed further (Table 2).

**Table 2:** Demographic and clinical characteristics of the TRS groups with and without admission

Variables	TRS with admission for FEP (*n* = 41)	TRS without admission for FEP (*n* = 38)	Statistic Values
*General information*			
Age (years)	41.6 [10.2]	42.1 [12.1]	*t* = −0.197, *P* = 0.844
Sex: Male/Female (n)	26/15	21/17	Chi = 0.544, *P* = 0.461
Follow-up duration (days)	6040.2 [3424.8]	6247.1 [4005.1]	*t* = −0.247, *P* = 0.805
Age at onset (years)	24.1 [7.5]	23.9 [9.2]	*t* = 0.122, *P* = 0.903
Duration before meeting TRS (days)	2538.9 [2640.0]	3885.9 [3511.6]	*t* = −1.994, *P* = 0.050
*Clinical index prior to treatment introduction*			
Premorbid social adjustment	4.27 [3.29]	5.18 [2.95]	*t* = −1.298, *P* = 0.198
DUP (months)	8.69 [17.6]	10.67 [15.39]	*t* = 0.512,
DUP Median	2.00	6.00	*P* = 0.610
Mode of onset: Acute/Insidious (n)	27/14	14/24	Chi = 6.65, ***P*** **= 0.010**
*Clinical index related to FEP*			
Duration of initial hospital admission (days)	169.3 [261.4]	0 [0]	*t* = 3.992, ***P*** **< 0.001**
GAF after FEP	53.2 [16.8]	46.8 [16.0]	*t* = 1.716, *P* = 0.090
GAF > 70 (n)	8	3	–
CP-dose for FEP treatment (mg)	654.4 [427.5]	467.9 [374.6]	*t* = 2.034, ***P*** **= 0.046**

*Abbreviations*: *CP* chlorpromazine, *DUP* duration of untreated psychosis, *FEP* first-episode psychosis, *TRS* treatment-resistant schizophrenia

#### Duration of initial hospital admission

In regard to the duration of initial hospital admission, the subgroup with a duration of initial hospital admission > 0 had a duration of 169 days on average, while the subgroup with a duration of 0 had a duration of 0 days by definition. Patients with TRS who were not admitted for FEP had lower GAF scores and smaller doses of antipsychotic at the end stage of FEP compared to patients with TRS who were admitted for FEP. Few patients achieved scores of 70 or more on the GAF: there were 8 such patients (19.5%) in the group of TRS patients with admission and 3 such patients (7.9%) in the group of TRS patients without admission.

#### Other measures

There were no significant differences in age, gender, age at onset or premorbid social functioning between the two TRS subgroups. While DUP was also not significantly different between the groups, the MoOs were significantly different: there were more patients with acute onset among the TRS patients with admission for FEP and more patients with insidious onset in the TRS patients without admission for FEP. There were no differences between the subgroups in the other parameters: admission times during the entire follow-up, or admission duration following FEP.

## Discussion

There were two main findings in the present study. 1) Although the TRS group showed slightly worse recovery from their FEP than the Non-TRS group in terms of GAF scores, there was no difference in the duration of initial hospital admission (defined as the period from treatment introduction to successful discharge, with successful discharge meaning 3 months of successful outpatient treatment) between the two groups, and 2) the patients in the TRS group experienced either one of two contrasting courses at the early disease stage: a) acute-onset psychosis, requiring hospital admission to introduce treatment and then a long hospital stay for FEP, or b) insidious onset without hospital admission and subsequent treatment with a relatively small dose of antipsychotics. The latter finding of dual early-stage courses helps to clarify the subtleties of the former finding that there were no differences in factors other than GAF at the end of FEP between the TRS and Non-TRS groups. That is, the fact that the FEP profile of the TRS group exhibited two distinct patterns dilutes the essential difference in the FEP profiles between the TRS and the Non-TRS groups. However, our results strongly indicate that poorer response or longer time course to improvement of either positive or negative symptoms following initiation of medications is a marker of risk for TRS.

No definitive definition of FEP duration has been established in either the clinical or research fields. Onset is generally defined as the appearance of positive symptoms, but it has proven difficult to determine the ending time point in a uniform manner across studies. This is partly because about 15% of patients under FEP do not respond to treatment [3, 4]. This is one of the reasons why, in the present study, we used the date of successful discharge from the hospital (not requiring subsequent readmission within 3 months) as an alternative endpoint of FEP. In addition, the medical insurance system of Japan encourages both a hospital admission shorter than 3 months and the avoidance of readmission within 3 months following discharge, by reducing reimbursement revenues unless these criteria are fulfilled. This peculiarity of the Japanese insurance system was also part of our rationale for using an absence of readmission for 3 months from the previous discharge as part of our definition of FEP duration in the present study.

Our present finding that patients who go on to develop TRS are more likely to have exhibited poor improvement during their FEP is in agreement with several clinical trials. Namely, several studies have reported that a poorer response to an initial pharmacological medication was closely related to a lower likelihood of recovery [13–17]. However, in the present study the TRS patients did not show an improvement of FEP symptoms extremely poorly at the 6th month compared to the Non-TRS patients (GAF score of 50.1 points vs. 61.0 points). This result was partly due to both the varied GAF scores in the TRS group (Fig. 3a) and the relatively low average GAF in the Non-TRS group, i.e., only 28.6% (*n* = 52) of the 182 subjects of the Non-TRS group achieved a GAF of > 70 points (Table 1).

In this study, the more important finding was that there were two distinctive subtypes of TRS patients. The TRS patients with admission for FEP had a GAF of only 53 points after 6 months, which is almost the same as their duration of initial hospital admission (169.3 days). That is, they were discharged from the hospital regardless of whether or not their improvement was sufficient after a half year of intensive medication and effortful care. On the other hand, the TRS patients without admission did not require hospital admission for their FEP and were treated under a relatively low dose of antipsychotics thereafter, implying that negative symptoms will be evident in this subtype. It is unlikely that more patients with intolerance to antipsychotics as a TRS subtype were included in the group of TRS patients without admission, since only four patients with intolerance to antipsychotics belonged to each of the two TRS subgroups. Therefore, higher susceptibility to extrapyramidal symptoms did not account for the low dose in the subgroup of TRS patients without admission. Furthermore, their DUPs, one of the best known predictors of treatment refractoriness, were similar or rather shorter than those of the Non-TRS group, indicating that there was no relationship between DUP and future development to TRS. Gender also showed no relation to TRS.

The two subtypes of TRS, however, were very similar in terms of the course following the initial hospital admission. That is, they did not differ in subsequent hospital admission times or durations, and both experienced gradual increases in antipsychotic dosage. Surprisingly, the duration from treatment introduction until meeting the TRS criteria was longer than previously thought. In this study, the duration was 3195 days on average (=8.75 years) or 2539 days (=6.96 years) in the TRS group with hospital admission and 3886 days (=10.65 years) in the TRS group without admission. The value in the TRS group without admission tended to be longer than that in the TRS group with hospital admission, but not significantly so (*p* = 0.070). These long durations until meeting the poor-responder criteria of TRS diagnosis may be related to long-term treatment with single or combined antipsychotic(s) from relatively few classes, which in turn might be related to the lack of availability of clozapine in Japan before 2009.

Lally and colleagues prospectively followed 246 patients with schizophrenia for 5 years and then studied their process of transition to TRS [7]. They found that 81 patients (33.7%) eventually fulfilled the TRS criteria, and 56 (70%) of these 81 patients showed no response to FEP treatment and directly met the TRS criteria (so-called “early TRS”), while the other 24 (30%) were improved by FEP medication but subsequently transitioned to TRS (so-called “late TRS”). Lally et al. speculated that the latter type may be related to dopamine supersensitivity psychosis.

Demjaha et al. reported the outcome of a 10-year follow-up of 434 patients in the same area (London) as in the study by Lally et al., presumably with a different cohort [8]. Among the 343 patients included in their final analysis, 74 patients (23%) were diagnosed as having TRS, 62 patients (84%) and 12 patients (16%) being diagnosed with early and late-TRS, respectively, which were quite similar to the results of Lally’s study. In summary, they concluded that the process of development to TRS could be divided into two such types of early disease course toward TRS. Since their studies differed from ours in terms of follow-up duration and clinical parameters (i.e., GAF and antipsychotic dose in our study vs. remission and clozapine treatment in theirs), a direct comparison between their results and our present findings is impossible. However, in our study there were 11 TRS patients who showed a good response to FEP treatment and realized a GAF score of 70 points or higher, which may correspond to the “late TRS” category in the studies of Lally et al. and Demjaha et al., indicating that the individual TRS subtypes overlap.

Our results suggest that there may be two additional subtypes within the early-TRS category, i.e., the category of patients who never achieve remission in early-stage treatment. The first group would consist of patients requiring a lengthy hospital stay with acute onset and profound positive symptoms, and second group would be those whose onset develops slowly with high negative symptoms. This strongly underscores the need for further study on the early process of TRS.

There are several limitations of the present study to consider, and caution should be taken in generalizing the results. Most importantly, the patients who dropped out of the analysis because they were transferred between hospitals and thus had insufficient available data made up a significant part of our cohort. The major reason for transferring to another hospital was the necessity of a longer hospital stay due to the severity of symptoms; if all TRS patients were included, the actual duration of initial hospital admission could have been longer than the calculated duration. This could constitute a form of selection bias. Second, the study design was retrospective, and considered only the duration of hospital admission, limiting complete clarification of the early process of schizophrenia. Therefore, a score of “0” for the initial hospital admission did not necessarily reflect the pathological process of schizophrenia, but might have been influenced by other factors such as the family’s support and residential area, etc. Third, since the overall data were derived from a real-world clinical setting, our results were influenced by physicians’ opinions on pharmacological medication as well as our domestic medical environment in Japan. For example, introduction of clozapine into clinical practice was delayed until 2009 in Japan. This delay could have led to continuous treatment in using the same regimen of antipsychotics without adjusting dosage or switching to a different class of agents even in patients who had failed to respond to previous treatments.

## Conclusions

In conclusion, among our cohort of patients with schizophrenia, those who progressed to TRS tended to show slightly less improvement during treatment for their FEP than those who did not progress to TRS. Two contrasting patterns of early-disease development appear to underlie the limited improvement at FEP: one pattern is acute onset with poor response of positive symptoms to treatment, and the other is insidious onset and profound negative symptoms.

## Abbreviations

CP-eq: Chlorpromazine-equivalent
CPMS: Clozaril Patients Monitoring Service
DUP: Duration of untreated psychosis
FEP: First episode psychosis
GAF: Global assessment of functioning
MoO: Mode of onset
TRS: Treatment-resistant schizophrenia
